# Atypical Obstructed Hemivagina and Ipsilateral Renal Anomaly (OHVIRA) Syndrome Without Hematocolpos in an Adolescent: A Case Report

**DOI:** 10.7759/cureus.117683

**Published:** 2026-10-09

**Authors:** Rayane Bekkaoui, Karima Sif Nasr, Salma Tabati, Laila Jroundi, Soukaina Allioui

**Affiliations:** 1 Radiology, Ibn Sina University Hospital, Rabat, MAR

**Keywords:** bicornuate bicollis uterus, herlyn-werner-wunderlich syndrome, müllerian anomaly, ohvira syndrome, renal agenesis

## Abstract

Obstructed hemivagina and ipsilateral renal anomaly (OHVIRA) syndrome is a rare congenital urogenital anomaly with variable Müllerian and renal manifestations. While the classic presentation includes postmenarchal pelvic pain and hematocolpos, incompletely obstructed forms may remain asymptomatic and be detected incidentally. We report the case of a 16-year-old girl with regular menstrual cycles and no prior gynecological symptoms who presented with acute urinary retention in the setting of a urinary tract infection. Ultrasonography showed non-visualization of the left kidney, a small cystic structure in the left renal fossa, and two uterine cavities. Computed tomography confirmed apparent left renal agenesis with compensatory hypertrophy of the right kidney and a probable involuted dysplastic renal remnant. Pelvic magnetic resonance imaging demonstrated a bicornuate bicollis uterus and a single non-distended vaginal canal, without hematocolpos, hematometra, or hematosalpinx. The overall findings were considered compatible with an atypical incompletely obstructed OHVIRA variant, although a small hemivaginal communication was not directly visualized. This case highlights the broad anatomical and clinical spectrum of OHVIRA syndrome and emphasizes the importance of evaluating the genital tract in adolescent girls with unilateral renal anomalies, even in the absence of typical gynecological symptoms.

## Introduction

Obstructed hemivagina and ipsilateral renal anomaly (OHVIRA) syndrome is an uncommon congenital urogenital malformation with a wide spectrum of uterine, cervical, vaginal, and renal abnormalities. Although the classic form is usually diagnosed following menarche because of dysmenorrhea, cyclic pelvic pain, or hematocolpos, incompletely obstructed variants may remain clinically silent and be discovered incidentally. Imaging, particularly magnetic resonance imaging, plays a central role in defining the complex anatomy and identifying associated complications [1].

We report an atypical OHVIRA presentation in a 16-year-old girl with no previous gynecological symptoms, in whom a bicornuate bicollis uterus and apparent left renal agenesis were incidentally identified during the investigation of acute urinary retention in the setting of a urinary tract infection. The absence of retained menstrual products and the inability to directly delineate a small hemivaginal communication illustrate the diagnostic challenges posed by incompletely obstructed variants.

## Case presentation

A 16-year-old adolescent girl with no significant medical or surgical history presented with acute urinary retention in the setting of a confirmed urinary tract infection. She reported regular menstrual cycles and had never experienced dysmenorrhea, cyclic pelvic pain, abnormal vaginal bleeding, or previous gynecological complaints.

In initial abdominopelvic ultrasonography performed during the urinary tract evaluation, the left kidney was not visualized in the renal fossa or pelvis and a small cystic structure within the left renal fossa was identified measuring 21x13 mm (Figure 1A). Pelvic ultrasonography demonstrated two distinct uterine cavities, raising suspicion of an associated Müllerian anomaly (Figure 1B).

**Figure 1:**
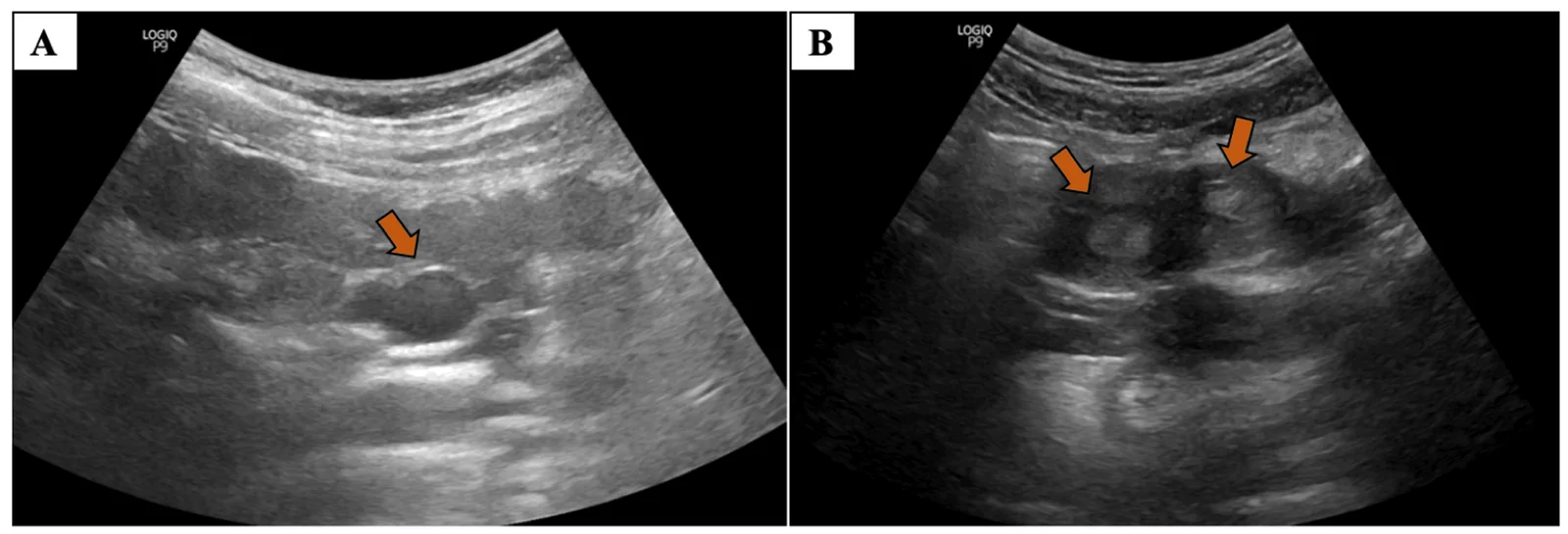
Initial Ultrasonographic Findings A: Ultrasound image of the left renal fossa showing non-visualization of normally developed renal parenchyma and a small cystic structure (arrow). B: Pelvic ultrasound image showing two distinct uterine cavities, raising suspicion of a Müllerian duct anomaly.

Subsequent abdominopelvic CT confirmed the absence of a normally developed left kidney, with compensatory hypertrophy of the right kidney (Figure 2A). The small cystic structure within the left renal fossa was considered most likely to represent an involuted dysplastic renal remnant (Figure 2B).

**Figure 2:**
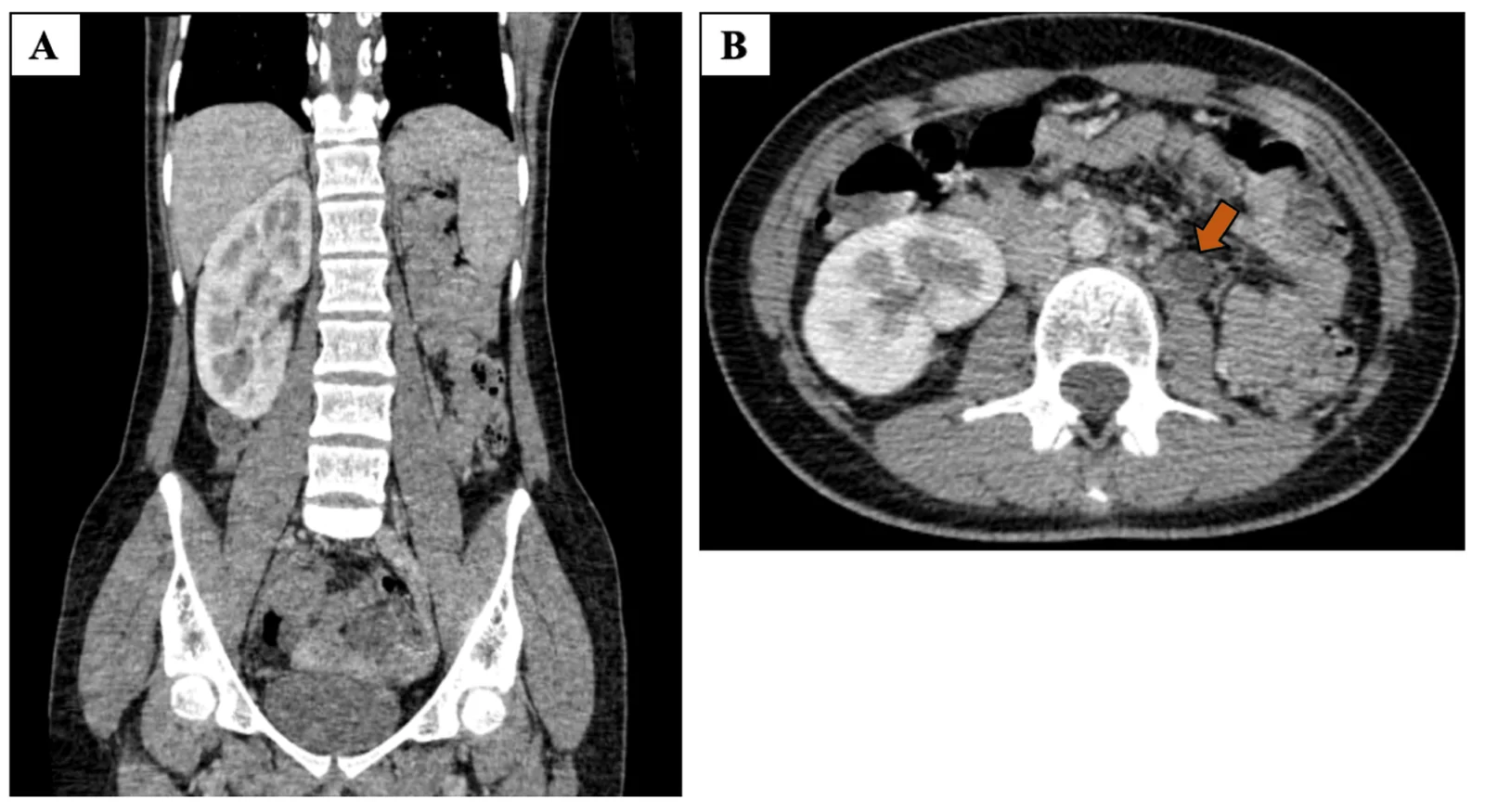
CT Findings of the Renal Anomaly A: Coronal contrast-enhanced CT image demonstrating absence of a normally developed left kidney and compensatory hypertrophy of the right kidney. B: Axial CT image showing a small well-defined cystic structure within the left renal fossa (arrow), considered most likely to represent an involuted dysplastic renal remnant.

Pelvic MRI demonstrated a deep external fundal cleft separating two divergent uterine horns, with two distinct endometrial cavities and duplicated cervices, consistent with a bicornuate bicollis uterus (Figure 3A, 3B). A single, non-distended vaginal canal was identified, without a clearly delineated longitudinal vaginal septum or hemivaginal distension. No hematometra, hematocolpos, hematosalpinx, or other retained blood products were present (Figure 3C). No ovarian endometrioma or other evidence of pelvic endometriosis was identified.

**Figure 3:**
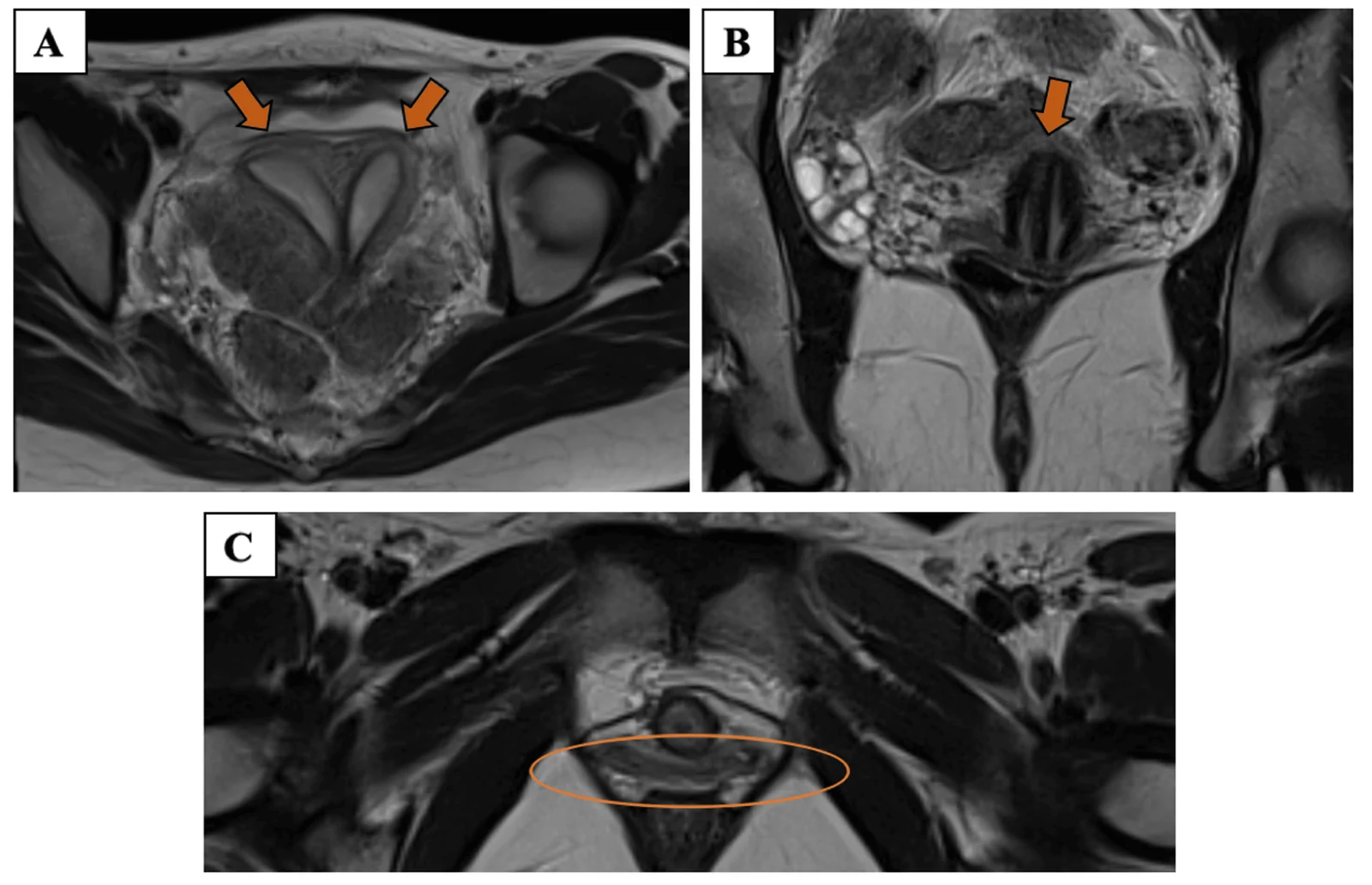
MRI Characterization of the Müllerian Duct Anomaly A: Axial T2-weighted MRI image demonstrating two divergent uterine horns with separate endometrial cavities, consistent with a bicornuate uterine configuration (arrows). B: Coronal T2-weighted image at the cervical level showing two distinct cervices (arrow), consistent with bicollis morphology. C: Axial T2-weighted image demonstrating a common, non-distended vaginal canal without hematocolpos.

The overall genitourinary findings were interpreted as an atypical, incompletely obstructed form of Herlyn-Werner-Wunderlich syndrome, also known as obstructed hemivagina and ipsilateral renal anomaly syndrome. The absence of hematocolpos was considered compatible with partial menstrual drainage through a subtle hemivaginal communication that was not fully delineated on imaging. The patient was referred to the gynecology department for further anatomical assessment and clinical follow-up.

## Discussion

Herlyn-Werner-Wunderlich syndrome, or OHVIRA syndrome, is a rare congenital urogenital anomaly classically characterized by uterine duplication, unilateral hemivaginal obstruction, and an ipsilateral renal anomaly, usually renal agenesis. It was first described by Purslow in 1922 and later characterized by Herlyn, Werner, and Wunderlich. The term OHVIRA was introduced to emphasize the association between vaginal obstruction and ipsilateral renal abnormality. Embryologically, abnormal mesonephric duct development disrupts both ipsilateral renal formation and adjacent Müllerian duct development, resulting in combined urinary and genital tract anomalies [1,2].

Although uterine didelphys with a completely obstructed hemivagina represents the classic form, OHVIRA encompasses a broader spectrum that includes bicornuate and septate uteri, cervical duplication or atresia, and variable degrees of vaginal obstruction. Complete hemivaginal obstruction generally becomes symptomatic soon after menarche due to retained menstrual blood, whereas incomplete obstruction may allow drainage and delay diagnosis [1,3].

The distinctive feature of our case was its incidental discovery during the investigation of acute urinary retention in the setting of a confirmed urinary tract infection, in the absence of any gynecological symptoms. This presentation is consistent with the clinically silent course that may occur in incompletely obstructed forms. Qi et al. reported later symptom onset in patients with incomplete obstruction, while Song et al. noted that some patients initially seek medical care for urinary problems and that others remain entirely asymptomatic and are diagnosed incidentally [1,4]. In our patient, the absence of hematocolpos, pelvic mass, and previous gynecological complaints supports an asymptomatic variant detected through an unrelated urinary presentation.

In a recent case series, Kim et al. described one patient with uterine and cervical duplication, communicating hemivaginas, and no hematocolpos or hematometra. In another patient, MRI failed to demonstrate the communication, which was identified only during speculum examination as a small defect in the vaginal septum with hemorrhagic drainage. These cases show that incomplete obstruction may occur without hematocolpos and that small septal communications can remain occult on MRI [5].

Diagnostic delay remains an important issue in OHVIRA syndrome. In a retrospective study of 164 patients, Song et al. found that 27 patients (16.5%) had initially been misdiagnosed, most commonly with pelvic abscess or ovarian cyst. Pelvic MRI before the definitive diagnosis was associated with a significantly lower risk of misdiagnosis, emphasizing its value in atypical or anatomically complex presentations [4].

Zhu et al. classified the syndrome according to whether the hemivagina is completely or incompletely obstructed. Type 2.1 corresponds to partial resorption of the vaginal septum, creating a small communication between the two hemivaginas and allowing menstrual drainage. Such patients may have normal menstruation, no hematocolpos or hematometra, and few or no gynecological symptoms. The absence of retained menstrual products and the normal menstrual history would be compatible with Zhu type 2.1 if a partially resorbed vaginal septum with a small communication is confirmed clinically [6].

Another notable finding was a small cystic structure within the left renal fossa, on the side of the absent kidney. Given its location, it was interpreted as a probable involuted dysplastic renal remnant associated with apparent left renal agenesis [7].

Recent evidence further illustrates the marked anatomical and clinical heterogeneity of OHVIRA syndrome. In a literature review of 1,673 patients, Liu et al. found that type 2.1, characterized by a small communication between the two hemivaginas, accounted for 39.2% of cases [8]. Systematic reviews have further highlighted the variability in clinical presentation and the importance of timely diagnosis and fertility-preserving management [9,10]. More recently, Bakhsh and Hamdoun emphasized the complementary role of ultrasonography as an initial imaging modality and MRI for detailed anatomical characterization and assessment of associated urinary tract anomalies [11].

This case illustrates the broad clinical and anatomical spectrum of OHVIRA syndrome by showing that it may be incidentally diagnosed in a gynecologically asymptomatic adolescent. It also highlights that uterine didelphys is not mandatory and that the absence of hematocolpos or a clearly visible hemivaginal communication does not exclude an incompletely obstructed variant [3-5]. Therefore, unilateral renal agenesis in adolescent girls should prompt systematic evaluation of the uterus, cervices, and vagina, even in the absence of pelvic pain or dysmenorrhea.

## Conclusions

OHVIRA syndrome should be considered when a Müllerian anomaly is associated with an ipsilateral renal abnormality, even in adolescents without dysmenorrhea, pelvic pain, or hematocolpos. Incompletely obstructed forms may be clinically silent and difficult to characterize fully on imaging. Careful MRI assessment, complementary gynecological evaluation, and appropriate follow-up are therefore important for defining the anatomy, facilitating timely management, and reducing the risk of obstructive or reproductive complications.
